# Stacking faults in β-Ga_2_O_3_ crystals observed by X-ray topography

**DOI:** 10.1107/S1600576718011093

**Published:** 2018-09-10

**Authors:** Hirotaka Yamaguchi, Akito Kuramata

**Affiliations:** a Advanced Power Electronics Research Center, National Institute of Advanced Industrial Science and Technology, 1-1-1 Umezono, Tsukuba, Ibaraki 305-8568, Japan; b Novel Crystal Technology Inc., 2-3-1 Hirosedai, Sayama, Saitama 350-1328, Japan

**Keywords:** X-ray topography, stacking faults, partial dislocations, slip planes

## Abstract

Planar defects in -oriented β-Ga_2_O_3_ wafers were studied using X-ray topography. These planar defects were rectangular with dimensions of 50–150 µm, and the X-ray topography analysis revealed that they were stacking faults (SFs) enclosed by a single partial dislocation loop on the  plane. The SF formation was found to be supported by a unique structural feature of the  plane as a slip plane; the  plane consists of close-packed octahedral Ga and O layers, allowing slips to form SFs. Vacancy arrays along the *b* axis in the octahedral Ga layer reduce the self-energy of the edge component in the partial dislocation extending along the *b* axis. It is speculated that the SFs occur during the crystal growth process for unknown reasons and then recover owing to elastic instability after initially increasing in size as crystal growth proceeds. Based on this analysis, a structural model for the SFs is proposed.

## Abstract 2

X-ray topography analysis reveals the presence of stacking faults in β-Ga_2_O_3_ wafers grown by the edge-defined film-fed growth method. These stacking faults are found to be associated with a Shockley-type partial dislocation loop on the basis of the contrast extinction rules determined by varying the value of the diffraction vector.

## 1. Introduction   

The monoclinic modification of gallium oxide (β-Ga_2_O_3_) is a promising material for power electronics applications owing to its superior physical properties, such as a wide electron energy band gap (Tippins, 1965 ▸) and high electric breakdown field (Higashiwaki *et al.*, 2012 ▸). β-Ga_2_O_3_ is the most thermally stable of the several polymorphs of Ga_2_O_3_ and high rates of crystal growth can be achieved using the melt growth technique. Recently, wafers cut from ingots grown by the edge-defined film-fed growth (EFG) method have become commercially available as substrates for electronic devices (Kuramata *et al.*, 2016 ▸), encouraging the further development of this material for electronic device applications. The first Ga_2_O_3_ metal-semiconductor field-effect transistors based on single-crystal β-Ga_2_O_3_ substrates were demonstrated by Higashiwaki *et al.* (2012 ▸).

Crystallographic defects such as dislocations and stacking faults (SFs) play a crucial role in device performance and reliability. The electrical properties of β-Ga_2_O_3_ diodes have been investigated using etch pit observations to study the relationship between defects and current leakage (Oshima *et al.*, 2017 ▸; Kasu *et al.*, 2017 ▸). Furthermore, the dislocations and hollow defects in β-Ga_2_O_3_ single crystals have been examined by X-ray topography (Nakai *et al.*, 2015 ▸), and the dislocations and twin structures have been analyzed by transmission electron microscopy (Ueda *et al.*, 2016 ▸). However, the detailed crystal structure of β-Ga_2_O_3_ containing defects has not yet been reported, and therefore the nature of these defects remains unclear. In our previous paper, we proposed a hypothetical slip system based on the crystal structure (Yamaguchi *et al.*, 2016 ▸). We also analyzed the dislocations using X-ray topography and identified the Burgers vectors and slip planes for some typical dislocations.

In this paper, we analyze planar defects on the 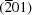 plane. The 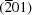 plane is the unique plane on which GaO_4_ tetrahedra and GaO_6_ octahedra are separately stacked, which enables partial dislocation by slip on the plane. The planar defects were found to be stacking faults enclosed by a partial dislocation loop.

## Crystallography of the (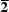
01) plane   

2.

### 2.1. Dislocations   

**Figure 1:**
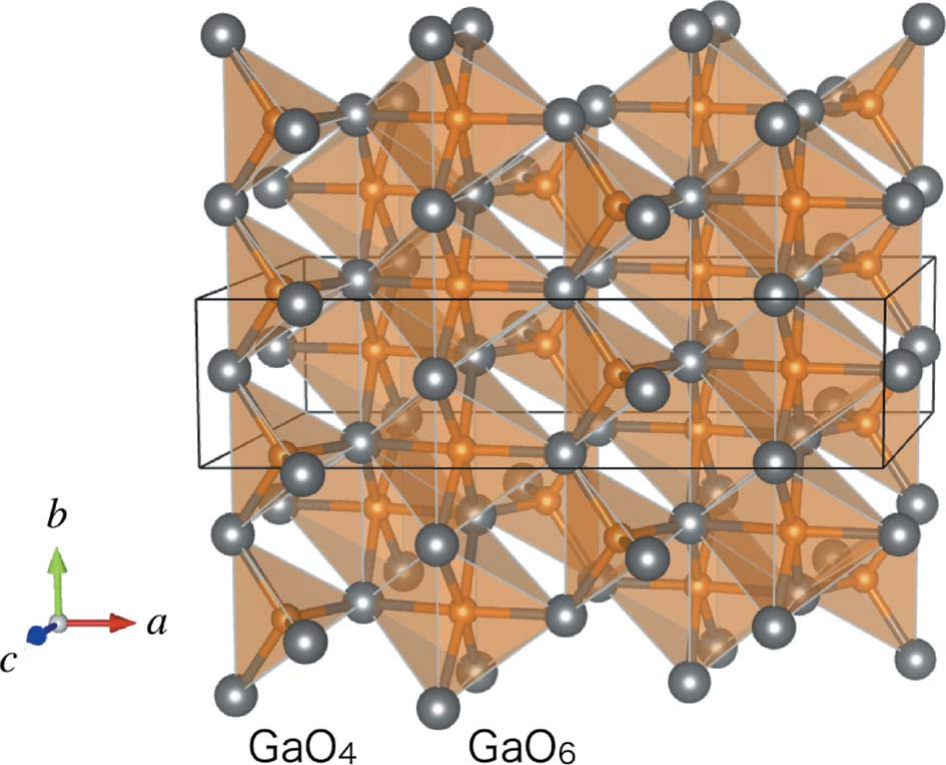
Crystal structure of β-Ga_2_O_3_ (figure prepared with *VESTA*; Momma & Izumi, 2011 ▸). The unit cell is depicted by thin lines.

**Figure 2:**
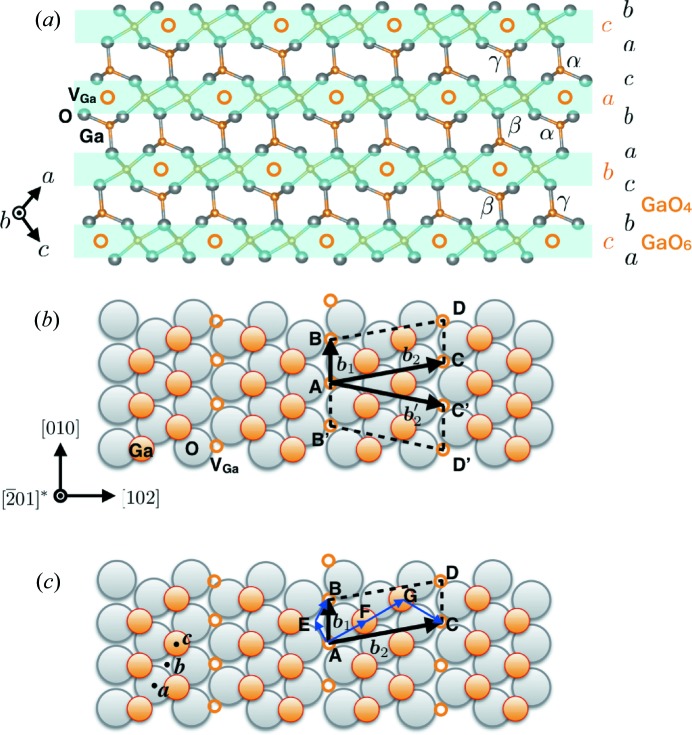
(*a*) Side view of the 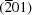 stacking structure. Atomic position indices *a*, *b* and *c*: in-plane positions of O ions and Ga ions in the GaO_6_ unit; α, β and γ: Ga ions in the GaO_4_ unit. (*b*) In-plane structure of octahedral Ga ions at the *c* positions on the underlying O ions at the *a* positions. The shortest unit translation vector, 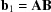, and the second shortest vectors, 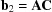 and 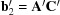, are shown. (*c*) Possible dissociation of dislocations with Burgers vectors of 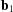 and 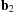 shown in (*b*). 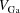 represents the vacancy site in the Ga layer in the GaO_6_ unit.

The crystal structure of β-Ga_2_O_3_ is monoclinic (space group 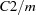) with the lattice parameters *a* = 12.23, *b* = 3.04, *c* = 5.80 Å, β = 103.7°, as depicted in Fig. 1 ▸ (Geller, 1960 ▸). The unit cell contains two different Ga–O structural units: GaO_4_ tetrahedra and GaO_6_ octahedra. As Geller described, ‘the oxygen ions are arranged in a ‘distorted cubic’ close-packed array’. The close-packed planes in β-Ga_2_O_3_, corresponding to the {111} planes of the face-centered cubic structure, create a distorted tetrahedron with faces formed by the 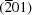, (101), 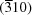 and 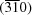 planes (Yamaguchi *et al.*, 2016 ▸). Among these planes, the 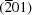 plane is unique in terms of its atomic arrangement; the tetrahedral GaO_4_ and octahedral GaO_6_ units are alternately stacked and are completely separated in a layer-by-layer manner (Fig. 2 ▸ *a*). As depicted in the figure, the stacking sequence of the O ions is 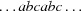, where *a*, *b* and *c* are the indices denoting in-plane positions. The possible Burgers vectors, which are the shortest and second shortest translation vectors in the 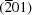 plane, are 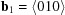 and 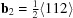 in both the tetrahedral and octahedral units. Fig. 2 ▸(*b*) shows an atomic model of the translational vector in the octahedral unit.

### 2.2. Dissociation   

If the total self-energy of a dislocation is reduced by decomposition of the original Burgers vector into a combination of shorter Burgers vectors, dissociation occurs. In the tetrahedral units, slip between widely spread layers, that is, the Ga–O units of the α–*a*, β–*b* and γ–*c* pairs in Fig. 2 ▸(*a*), is difficult to directly dissociate into partials, which is similar to the ‘shuffle set’ in a diamond cubic lattice (Hirth & Lothe, 1982 ▸). On the other hand, in the octahedral Ga units, the Ga ions are positioned in a close-packed arrangement between the O layers; that is, the Ga ions at the *c* positions are located between the O ions at the *a* and *b* positions on the underlayer and overlayer of the Ga layer, respectively, the Ga ions at the *b* positions are located between the O ions at the *c* and *a* positions, and so on. ‘Glide-set’ dislocations as in the diamond cubic lattice are, therefore, expected in the GaO_6_ units on the 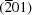 plane in β-Ga_2_O_3_. As shown in Fig. 2 ▸(*c*), the Burgers vector 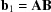 (displacement between the *c* positions) dissociates into 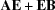, where displacement occurs *via* a *b* position, that is, 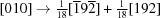. We refer to this type of dissociation as ‘Shockley partials’ after the case of the diamond cubic lattice.

We next consider another type of dissociation, 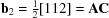, the norm of which is larger than that between the neighboring *c* positions. This displacement can be composed of the three shortest 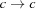 displacements, for example, 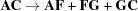, where 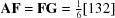 and 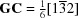. This type of dissociation is analogous to the model proposed by Kronberg (1957 ▸) for basal slip in sapphire (α-Al_2_O_3_). As the longer period of the Ga ions arises from a superlattice with regularly aligned empty sites on the Ga layer, we refer to this type of dissociation as ‘superlattice partials’.

## 3. Experiments   

The samples used in this study were 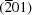-oriented wafers with a diameter of two inches and a thickness of 680 µm cut from ingots grown by the EFG method in 2015. X-ray topography experiments using synchrotron radiation from a bending magnet were performed at beamlines BL-3C and BL-20B at the Photon Factory, Institute of Materials Structure Science, High Energy Accelerator Research Organization (Tsukuba, Japan), and beamlines BL09 and BL15 at the SAGA Light Source (SAGA-LS), Kyushu Synchrotron Light Research Center (Tosu, Japan). The diffraction geometry was Bragg-case asymmetric diffraction with a low incident angle. The X-ray wavelength was selected using the double-crystal Si 111 monochromator such that the incident angle was optimized for each diffraction vector (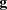). The X-ray topographs were recorded on nuclear emulsion plates (Ilford L4).

## 4. Results   

**Figure 3:**
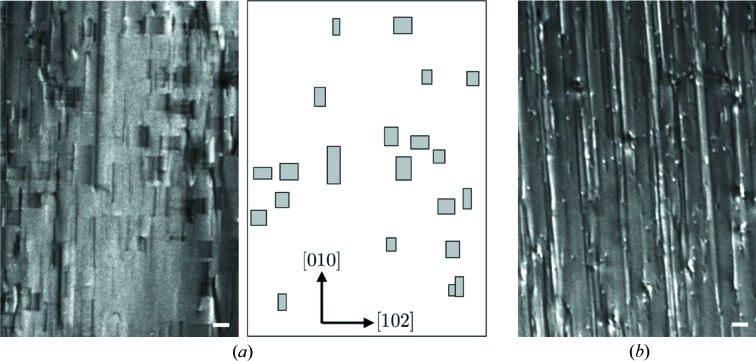
X-ray topographs of a 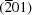-oriented wafer obtained with (*a*) 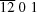 and (*b*) 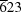 reflections. Some apparent SFs are schematically depicted on the right-hand side of (*a*). The scale bars correspond to 100 µm.

In the X-ray topographs of the 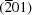 wafers, rectangular defects were observed at appropriate values of 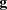, in addition to several dislocations, as shown in Fig. 3 ▸; the rectangular defects were observed in the X-ray topograph measured with the 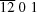 reflection but not in that measured with the 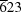 reflection. These defects are widely scattered throughout the wafer, although their density varies with the location. The finding that the rectangular defects were visible or invisible depending on 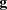 indicates that they are crystallographic defects in the crystal. As the aspect ratios of the rectangles measured with different 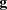 values were almost unchanged, the defects are presumed to lie parallel to the wafer surface 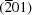.

Another feature of the planar defects is that they are isolated, with no connections to any dislocations. The planar defects are therefore considered to be SFs enclosed by a single partial dislocation loop on the 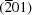 plane, indicating that the fault vector of the SF should be identical to the Burgers vector of the partial dislocation loop.

**Figure 4:**
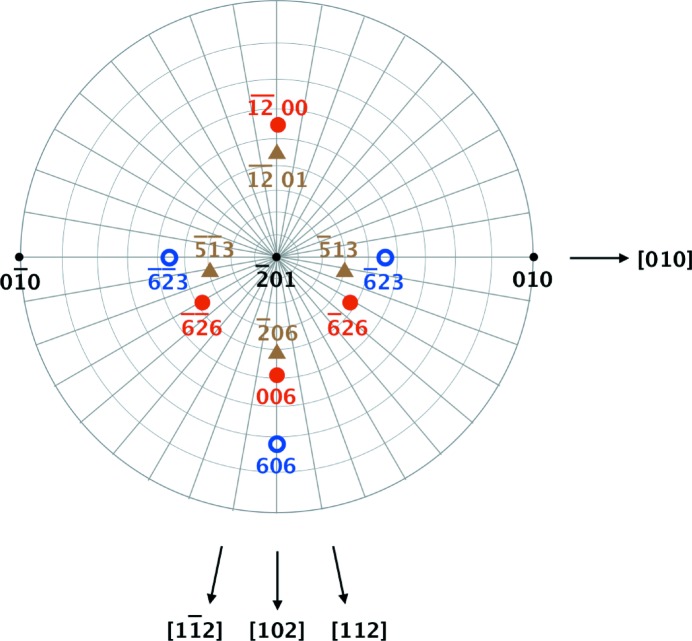
Pole figure for 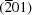. Diffraction indices are shown with the symbols indicating that both Shockley and superlattice SFs were invisible (open circles) or visible (filled triangles), or that Shockley SFs were visible but superlattice SFs were invisible (filled circles). Some directions in the real lattice are indicated.

The contrast of SFs in X-ray topography is associated with the relative change in the structural factors between the matrix and SF regions (Whelan & Hirsch, 1957 ▸; Kohra & Yoshimatsu, 1962 ▸). We cut a crystal along a particular plane, and shifted the upper part parallel to the plane with a vector 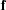, the magnitude of which was smaller than the unit lattice vector, relative to the lower part. We set the structural factor for the reflection 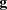 in the lower part (matrix) as 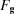, such that the factor in the upper part (SF) was 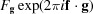. When 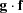 ≠ (integer), the structural factors in the two parts separated by the SF were different, and the SF exhibited contrast in the diffraction intensity. To examine the nature of each SF, that is, whether they were Shockley or superlattice partials, we compared the appearances of the SFs in the X-ray topographs obtained for several values of 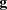. The conditions under which the SFs appeared are summarized in Table 1 ▸. The results from the X-ray topographs measured with 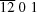 and 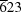 reflections (Fig. 3 ▸) were found to be consistent with the contrast extinction rules regardless of the type of dissociation, that is, Shockley or superlattice partials. The 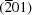 pole figure indicating the diffraction indices with which X-ray topographs were acquired in this study is presented in Fig. 4 ▸.

**Figure 5:**
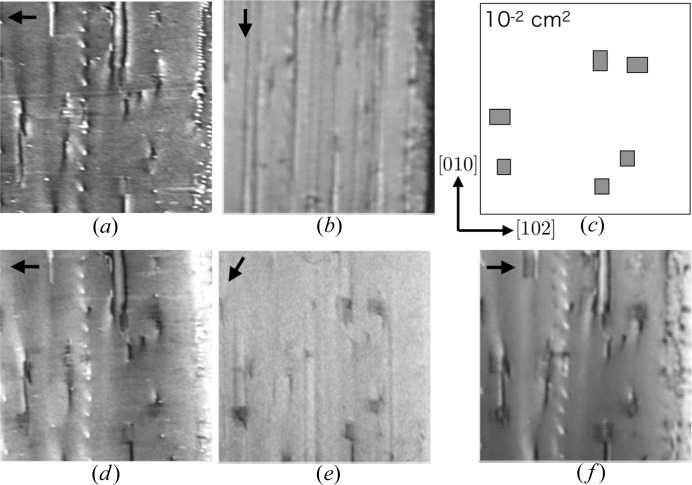
X-tay topographs obtained using various values of 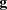: (*a*) 606, (*b*) 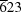, (*d*) 006, (*e*) 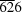 and (*f*) 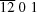 reflections. (*c*) Schematic view of planar defects in the topographs (*d*)–(*f*). The arrows indicate the direction of X-ray incidence. The X-ray wavelength for each topograph is listed in Table 1 ▸.

The experimental results relating to the 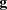 dependence of the SF appearance are summarized in Table 1 ▸(*a*), and some representative X-ray topographs are presented in Fig. 5 ▸. Almost all of the observed SFs exhibited the same 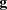 dependence and no obvious exceptions were found. The contrast of the SFs in the X-ray topographs was sensitive to the diffraction conditions. The existence and shape of each SF were confirmed by comparing a series of X-ray topographs acquired with small (up to 30′′) stepwise variation of the X-ray incident angle. Fig. 5 ▸ shows single shots selected from a series of X-ray topographs for each 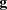, and therefore not all of the SFs depicted in Fig. 5 ▸(*c*) are readily apparent in Figs. 5 ▸(*d*)–5 ▸(*f*). Thus the SFs observed in this study were determined to be those associated with Shockley partials. As shown in Fig. 5 ▸, six SFs were detected in a 1 mm square, meaning that the SF density in this area can be roughly estimated as 600 cm^−2^. The characteristics of the SFs can be summarized as follows:

(1) They are rectangular and observed parallel to the surface 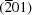 plane.

(2) One pair of sides elongates in the 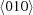 direction, and the other pair of sides is in the perpendicular direction, 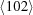.

(3) They are isolated SFs associated with Shockley partials.

We briefly comment about some typical dislocations found in Fig. 5 ▸. The arrays of points with bright contrast in (*a*), (*d*) and (*f*) are dislocations running through the wafer surface although their Burgers vectors are unidentified yet. We also find some lines running in the vertical direction in (*b*) and (*e*). They can be identified as screw dislocations with the Burgers vector 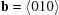 from the 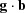 relation.

## 5. Discussion   

As explained in §2, the close-packed stacking structure of the 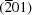 plane enables slip between the glide-set planes. During the analysis of the SFs, we considered the possibility of the superlattice partials because they can be derived from the close-packed structure. The superlattice partials were, however, disproved experimentally by the contrast extinction rules depending on 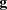. The other plausible possibility for the SFs is that they are associated with prismatic dislocation loops by the insertion or extraction of partial atomic layers. In this case, the fault vector would contain a component perpendicular to the 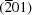 plane, resulting in different appearance rules from those observed.

**Figure 6:**
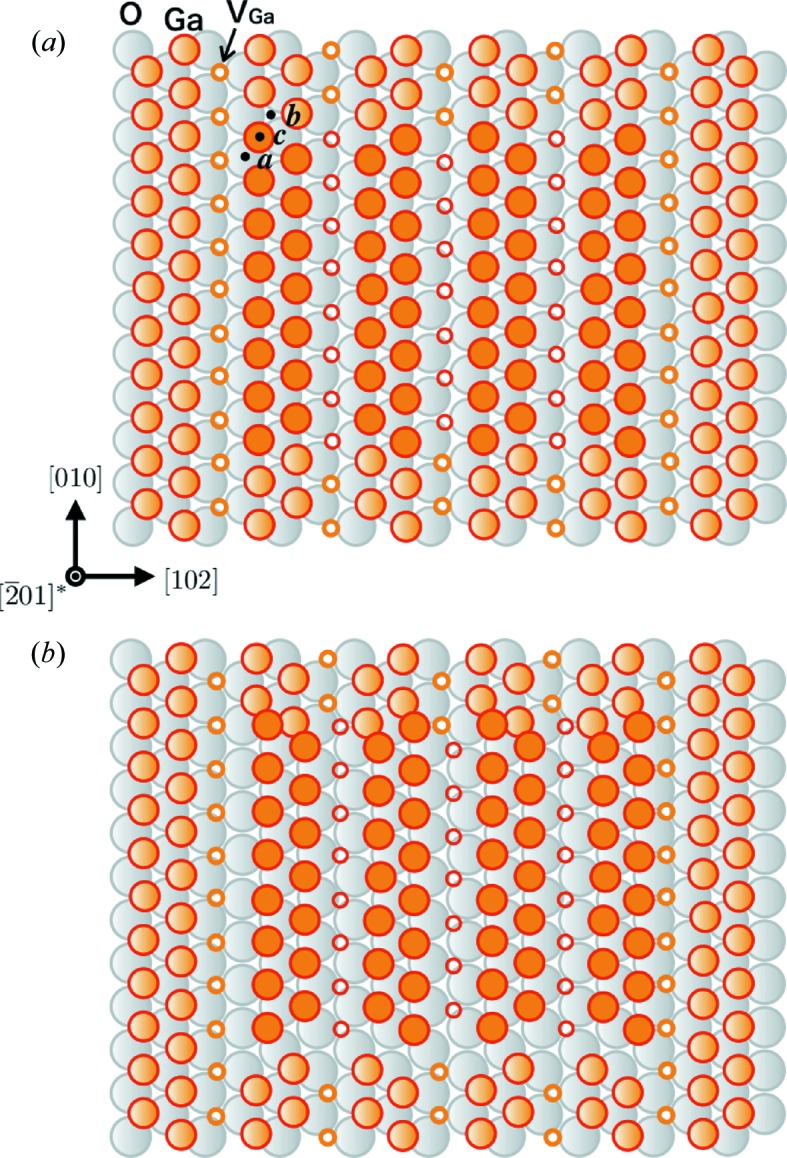
The proposed SF model. (*a*) Before slip: O ions lie in the underlayer and Ga ions occupy the *a* and *c* positions, respectively. (*b*) After slip: Ga ions in the central region have slipped from the *c* to the *b* positions to become enclosed by a Shockley partial dislocation loop.

Note the vacancy arrays running along the *b* axis in the octahedral Ga layers (Fig. 2 ▸). If a glide-set slip between the octahedral Ga and O layers occurred with the boundary along the vacancy array, it would reduce the self-energy of dislocation along the *b* axis. This is because the elastic compressive or tensile stress of the edge component is reduced by the gap and no dangling bonds occur at the partial dislocation. The Ga—O bonds exhibit rotation by 60° and no polarization. The proposed SF model based on this consideration is illustrated in Fig. 6 ▸. When the highlighted region in the original structure (Fig. 6 ▸ *a*) slips in the octahedral Ga layer, the Ga ions at the *c* positions slip with the boundary along the vacancy arrays parallel to the *b* axis over the underlying O ions at the *a* positions. The possible destination sites of the Ga ions after the slip are the *b* positions. As they slip 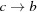, the resulting SF is as depicted in Fig. 6 ▸(*b*).

The wafers were cut from ingots grown using the EFG method with the *b* axis as the growth direction. The origin of the SFs is currently unclear. The SFs would be expected to elongate as the crystal grows along the *b* axis, which would increase their elastic energy. Eventually, the elastic energy would reach a critical value and the SF would then terminate to afford the rectangular shape observed in this study. The observed SF dimensions, 50–150 µm, may be typical values determined by the elastic energy. As no dislocations associated with the SFs were observed, SFs originating from dissociation of a dislocation are not considered here.

**Figure 7:**
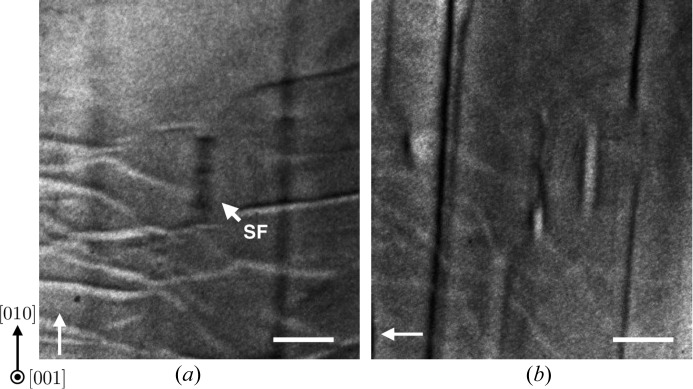
Comparison of X-ray topographs from a (001)-oriented wafer acquired with (*a*) 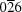 and (*b*) 606 reflections. The scale bars correspond to 0.1 mm. The arrows indicate the direction of X-ray incidence.

SFs were also observed in X-ray topographs of a (001)-oriented wafer. Fig. 7 ▸ shows a comparison of images taken with 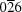 and 606 reflections. An SF was observed under the former but not the latter conditions, which is consistent with the contrast extinction rules for Shockley SFs as summarized in Table 1 ▸(*b*). The SF was as wide as 30 µm, which is less than the typical width observed in the 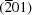 wafers. Upon taking into account the crossing angle between the 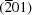 and 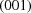 planes, which is 50° toward the left-hand side in Fig. 7 ▸(*a*), the apparent width of the SF on the 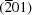 plane shrinks by a factor of cos50°. Although the true width of the SF is unknown, the SF observed in the (001)-oriented wafer can be regarded as originating from a Shockley partial dislocation loop on the 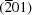 plane, which is identical to that observed in the 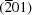-oriented wafers.

The SFs were found to be scattered in the wafers and possibly created by the crystal growth and/or subsequent cooling processes, although the underlying mechanism remains unclear at present. The observation of SFs in this study partially supports our hypothetical slip system, 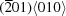 (Yamaguchi *et al.*, 2016 ▸). This means that the SFs may be glissile, potentially leading to enlargement of the SFs by stress or other factors. As demonstrated for SiC diodes, glissile partial dislocations affect degradation in forward-bias operations owing to the recombination–enhanced dislocation glide effect (Maximenko *et al.*, 2005 ▸). The SFs in β-Ga_2_O_3_ wafers, therefore, should be eliminated for electronic device applications.

## 6. Conclusion   

X-ray topography analysis revealed the presence of SFs in β-Ga_2_O_3_ wafers grown by the EFG method. These SFs were found to be associated with a Shockley-type partial dislocation loop on the basis of the appearance rules determined by varying the value of 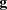 used during the X-ray topography. An SF model was proposed by considering the close-packed stacking structure of the GaO_6_ octahedral units in the 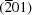 plane, on the assumption that the partial dislocation occurs at the gap in the Ga layer along the *b* axis. The occurrence of crystallographic defects such as dislocations and SFs is an important issue for the fabrication of electronic devices. Further studies of these SFs should be performed to elucidate their generation mechanism during the crystal growth process, their development during device processing and their effects on the electronic properties of the final devices, as well as the detailed characteristics and properties of SFs and other crystallographic defects.

## Figures and Tables

**Table 1:** Conditions for the appearance of SFs in (*a*) 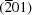 and (*b*) 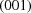 wafers, 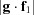 and 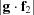, for several reflections 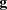 used in this study The fault vectors 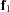 and 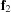 correspond to the Shockley and superlattice partials, respectively. The Appearance column shows whether the SFs were observed (Yes) or not observed (No). The λ column shows the X-ray wavelengths used for the topography, and the 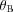 and α columns show the Bragg angles and the angles between the Bragg plane and the crystal surface, respectively.

	**g**	|**g** · **f**_1_|	|**g** · **f**_2_|	Appearance	λ (nm)	θ_B_ (°)	α (°)
(*a*)	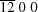	2/3	2	Yes	0.17	59.1	53.8
	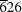	4/3	2	Yes	0.11	43.5	38.2
	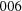	2/3	2	Yes	0.15	53.0	49.9
	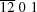	5/9	5/3	Yes	0.15	47.5	43.7
	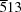	5/9	2/3	Yes	0.165	34.1	29.6
	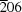	5/9	5/3	Yes	0.15	51.1	40.9
	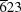	1	1	No	0.17	51.3	45.7
	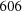	1	3	No	0.151	75.4	72.4

(*b*)	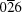	1/3	1	Yes	0.10	38.7	31.7
	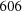	1	3	No	0.10	39.9	22.5
